# Subcellular Localization of Class II HDAs in *Arabidopsis thaliana*: Nucleocytoplasmic Shuttling of HDA15 Is Driven by Light

**DOI:** 10.1371/journal.pone.0030846

**Published:** 2012-02-17

**Authors:** Malona V. Alinsug, Fang Fang Chen, Ming Luo, Ready Tai, Liwen Jiang, Keqiang Wu

**Affiliations:** 1 Institute of Plant Biology, College of Life Science, National Taiwan University, Taipei, Taiwan; 2 Key Laboratory of Plant Resources Conservation and Sustainable Utilization, South China Botanical Garden, Chinese Academy of Sciences, Guangzhou, China; 3 School of Life Sciences, Centre for Cell and Developmental Biology, The Chinese University of Hong Kong, Shatin, New Territories, Hong Kong, China; National University of Singapore, Singapore

## Abstract

Class II histone deacetylases in humans and other model organisms undergo nucleocytoplasmic shuttling. This unique functional regulatory mechanism has been well elucidated in eukaryotic organisms except in plant systems. In this study, we have paved the baseline evidence for the cytoplasmic and nuclear localization of Class II HDAs as well as their mRNA expression patterns. RT-PCR analysis on the different vegetative parts and developmental stages reveal that Class II HDAs are ubiquitously expressed in all tissues with minimal developmental specificity. Moreover, stable and transient expression assays using HDA-YFP/GFP fusion constructs indicate cytoplasmic localization of HDA5, HDA8, and HDA14 further suggesting their potential for nuclear transport and deacetylating organellar and cytoplasmic proteins. Organelle markers and stains confirm HDA14 to abound in the mitochondria and chloroplasts while HDA5 localizes in the ER. HDA15, on the other hand, shuttles in and out of the nucleus upon light exposure. In the absence of light, it is exported out of the nucleus where further re-exposition to light treatments signals its nuclear import. Unlike HDA5 which binds with 14-3-3 proteins, HDA15 fails to interact with these chaperones. Instead, HDA15 relies on its own nuclear localization and export signals to navigate its subcellular compartmentalization classifying it as a Class IIb HDA. Our study indicates that nucleocytoplasmic shuttling is indeed a hallmark for all eukaryotic Class II histone deacetylases.

## Introduction

Histone acetylation has been known to induce an open chromatin configuration leading to transcriptional activation while deacetylation stimulates chromatin condensation triggering transcriptional quiescence. Plant histone deacetylases (HDA or HDACs) are classified into three distinct families namely RPD3/HDA1 superfamily, Sirtuin family, and the HD2 family which is unique in plants [1], [2], [3].

Twelve out of the eighteen known HDAs in Arabidopsis belong to the RPD3/HDA1-like histone deacetylase superfamily, which is further subdivided into three classes namely Class I, II, and IV. Recent phylogenetic studies by Alinsug *et al.* [4] have identified HDA10 and HDA17 in addition to HDA6, HDA7, HDA9, and HDA19 as Class I HDA based on sequence homology. Although HDA8 and HDA14 are only represented in plants, they exhibit higher sequence similarity with the conserved histone deacetylase domains of HDA5, HDA15, and HDA18, members of the Class II HDAs while HDA2 is classified as Class IV.

Among the RPD3/HDA1-like superfamily HDAs in Arabidopsis, HDA6, HDA19, and HDA18 have been well elucidated to play crucial roles in plant development and environmental stress response and display tissue-specific expression [5]–[11], [3]. HDA6 is the most extensively studied plant histone deacetylase acting as a global repressor involved in flowering, freezing tolerance, ABA and salt stress response, senescence, repression of embryonic properties, JA pathway, and establishment of nucleolar dominance [12]–[16], [10]. Similarly, HDA19 is a global repressor in embryonic and flower development, ABA and abiotic stress response, light-responsive gene expression, JA and ethylene signaling, and regulates basal defense via interaction with WRKY transcription factors [10], [5], [6], [17], [8], [18], [11]. HDA18, on the other hand, had been implicated in root epidermal patterning [19].

Previous studies by Finkemeier *et al.* [20] and Wu *et al.* [21] elaborated on the reversible acetylation of cytoplasmic proteins in Arabidopsis indicating that histones are not the only proteins being acetylated & deacetylated. A substantial proportion of these cytoplasmic proteins are involved in photosynthesis and central metabolism where the deacetylation of rubisco and phosphoglycerate kinase using human Sirt3 lead to a significant increase in their catalytic activity. However, the use of human Sirt3 as a deacetylase may exhibit certain specificities towards plant Lys acetylation sites. Thus, the observed potency may have been underestimated had a plant specific deacetylase been used. Unfortunately, none of the 18 known histone deacetylases in Arabidopsis have been identified to be cytoplasmic since HDA6 and HDA19 were established to be exclusively nuclear. In rice, OsSirt2b was found to localize in the mitochondria, OsHDAC6 in chloroplasts, and OsHDAC10 in both chloroplast and mitochondria although the localization of their respective homologues in Arabidopsis namely, SRT2, HDA2, and HDA14, still remains to be elucidated [22].

Class II histone deacetylases in humans and other metazoans have been well described to undergo nucleocytoplasmic shuttling [23]–[26]. This functional regulatory mechanism renders Class II HDACs to be active as histone deacetylases while inside the nucleus and inactive when exported out into the cytoplasm. Class II HDAs generally contain a nuclear localization signal (NLS) at the amino terminal and a nuclear export signal (NES) near the carboxyl end. However, mammalian Class II HDACs are classified further into Class IIa (HsHDA504, HsHDA505, HsHDA507, and HsHDA509) and Class IIb (HsHDA506 and HsHDA510) [24]. Class IIa HDACs are dependent on 14-3-3 binding to translocate into the cytoplasm while Class IIb HDACs rely on their strong NES and NLS for nuclear import and export. Although most of these Class II HDACs remain inactive in the cytoplasm, HDAC7 and HDAC6 continue to play versatile roles such as mitochondrial proteins implicated in apoptosis, SUMO E3 ligases, and target several cytosolic substrates such as tubulin, cortactin, Hsp90, β- catenin, and peroxiredoxin [27]–[38].

While inside the nucleus, Class IIa HDACs form complexes with MEF2, a subfamily of MADS-box transcription factors, which generally function as transcriptional repressors. They are likewise phosphorylated by Ca^2+^ dependent calmodulin kinase prior to binding with 14-3-3 proteins. This phosphorylation-dependent binding of 14-3- 3 to the N terminus of Class II HDACs masks the arginine/lysine-rich motif NLS which simultaneously unmasks a latent NES near its C terminus. Once phosphorylated, they are inactivated as a histone deacetylase and chaperoned out of the nucleus [26], [39]. When proper environmental signals are cued, these HDACs translocate back into the nucleus, dephosphorylated via binding with MEF2 transcription factors, and activated as histone deacetylases [23], [28].

In general, 14-3-3 proteins are highly conserved, multifunctional regulatory proteins, which have been implicated in the modulation of distinct biological processes by phosphorylation-dependent protein binding interactions [40]. There are 13 known isoforms in Arabidopsis, which are divided into two phylogenetic groups [41], [42]. Functional diversity and redundancy among these isoforms remain an open debate [43], [44]. Comparative interactomic studies by Paul and his colleagues [45] reveal highly conserved 14-3-3 interactions between humans and plants. Proteomic profiling using tandem affinity purified 14-3-3 complexes in Arabidopsis suggested the high potential of 14-3-3s to heterodimerize *in vivo* [46]. To date, there are more than 300 known client targets of plant 14-3-3s and most these interacting clients are involved in primary metabolism, ion homeostasis, and hormone signaling including ABA, BR, and GA [45], [46], [40], [47]–[54].

On the other hand, Class IIb HDACs in humans generally contain double domains, manifest tissue specificity, predominate in the cytoplasm, and target the deacetylation of histones, tubulin, Hsp90, cortactin, β-catenin, and peroxiredoxin [24], [23], [55]–[57], [34], [32], [37],[38]. In human HDAC6, it has been shown that its two catalytic domains function independently as proven by site directed mutagenesis of its two HDAC domains [58]. Consequently, the separation of these domains commences into the obliteration of its enzymatic activity [24]. On the other hand, the C-terminal domain of HDAC10 lacks an active residue required for its enzymatic activity. However, its interaction with the functional N-terminal domain renders it active as a histone deacetylase [59].

Based on sequence analyses of plant Class II HDAs in our previous study [4], not all of these five HDAs contain both NLS and NES. However, they all have conserved Ser and Thr residues, which can be potential phosphorylation sites for 14-3- 3 binding. Moreover, HDA18 contains a double HDAC domain, which is comparable to Class IIb HDACs but its conserved histone deacetylase domain is 54% similar to HDA5. In addition, human HDAC6 has a cysteine- and histidine-rich domain called ZnF-UBP which parallels with the ZnF-RanBP of HDA15. Still, the localization of plant Class II HDAs is unknown and their potential to undergo nucleocytoplasmic shuttling remains elusive.

Studies on plant Class II HDAs are scarce and pertinent information on their subcellular compartmentalization and specific expression patterns would provide significant insights on their potential function and active roles in plant development. Based on our findings, RT-PCR analysis on the different vegetative parts and developmental stages of Arabidopsis plants reveals that Class II HDAs are ubiquitously expressed throughout all tissues with minimal developmental specificity. Moreover, stable and transient expression assays using HDA-YFP/GFP fusion constructs indicate cytoplasmic localization of HDA5, HDA8, and HDA14 further suggesting their potential for nuclear transport and deacetylating organellar and cytosolic proteins. Organelle markers confirm HDA14 to abound in the mitochondria and chloroplasts while HDA5 localizes in the ER. HDA15, on the other hand, shuttles in and out of the nucleus upon light exposure. In the absence of light, it is exported out of the nucleus while further re-exposition into light treatments signals its nuclear import. Taken together, this provides the final piece of the puzzle indicating that nucleocytoplasmic shuttling is indeed a hallmark for all eukaryotic Class II histone deacetylases.

## Results

### 1. Expression patterns and localization of Class II HDAs

**Figure 1:**
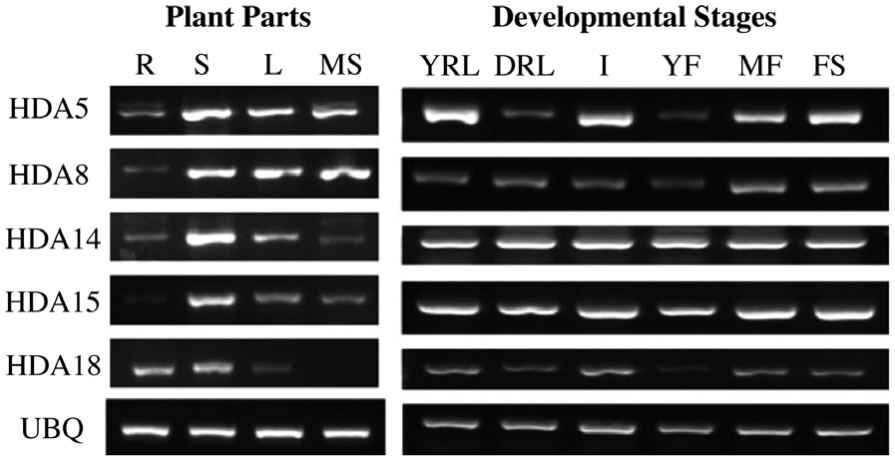
Class II HDA mRNA expression. Expression patterns of Class II HDAs in different vegetative organs and developmental stages were assessed using RT-PCR with ubiquitin (UBQ) as loading control. Legend: R roots, S stem, L leaves, MS mature seeds, YRL young rosette leaf, DRL developed rosette leaf, I inflorescence, YF young flower, MF mature flower, FS flowers and siliques.

mRNA expressions of Class II HDAs were assessed in Col-0 using different vegetative organs and whole plants at varying developmental stages. As illustrated in Figure 1, Class II HDA transcripts were broadly expressed in all the organs and developmental stages indicating that they may play an active role in the plant's overall growth and development. In comparison to all the plant parts, the stems elicited the strongest expression in all the Class II HDAs. Moreover, only HDA18 was abundantly expressed in roots but remained minimal for HDA14, HDA8, and HDA5. Although HDA18 appears to be actively expressed in roots and stems, they were barely detected in the leaves and mature siliques. On the other hand, transcript levels of HDA15 were prominently detected at the upper shoot parts but not in the roots.

At varying developmental stages, HDA5, HDA8, and HDA18 exhibited developmental specificity while HDA14 and HDA15 were homogenously yet strongly expressed all throughout the developmental stages. The expression of HDA5 is kept at a minimum during developed rosette leaf and young flower. HDA8 is weakly expressed from young rosette leaf to young flower but remains abundant thereafter. In addition, HDA18 is equally expressed throughout development but remains low during developed rosette leaves and undetected during young flower stage.

### 2. HDA5, HDA8, and HDA14 are localized in the cytoplasma

**Figure 2:**
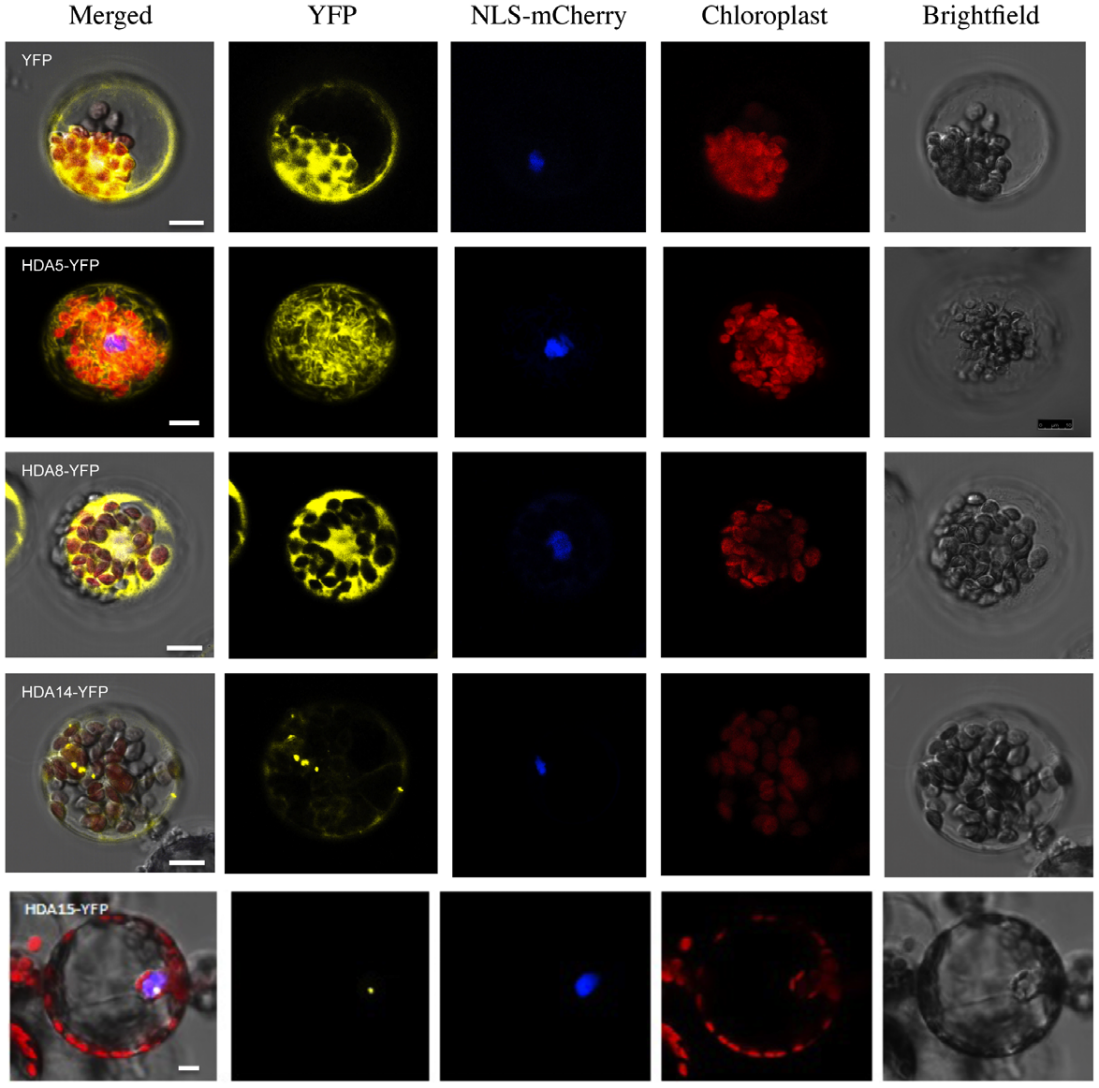
Subcellular localization of Class II HDAs. Protoplast transient expression using HDA-YFP fusion constructs were used to determine the subcellular localization of Class II HDAs. HDA5, HDA8, and HDA14 exhibited cytoplasmic localization while HDA15 concentrates inside the nucleus. VirD2NLS fused with mCherry was used as a nuclear marker. Scale bars were calibrated to 10 µm.

To determine the subcellular localization of Class II HDAs, HDA-YFP constructs with the YFP fused at the C terminal of the HDAs were used for transient expression in protoplast. As exhibited in Figure 2, HDA5 displayed prominently cytoplasmic concentrations with partial overlaps inside the nucleus. Strong HDA8- YFP signals were detected in the cytoplasm and embedded near the nucleus while HDA14 exhibited multiple small granular spots along the cytoplasma. On the other hand, HDA15 was exclusively nuclear.

In concurrence with this, another set of constructs were made fusing the GFP at the N-terminal of the HDAs which may mask some of the organelle targeting sequences imprinted along the histone deacetylases. Apparently, these GFP-HDA fusion constructs yielded similar results with the HDA-YFP set. HDA5 was prominently cytoplasmic lining through the core across the nucleus. HDA8 was found to abound in the cytoplasm enveloping the chloroplasts. HDA14 was evidently cytoplasmic with big and small granular spots floating along the cytoplasma. On the other hand, HDA15 was clearly restricted in the nucleus with strong signals emanating from the nucleolus (Figure S1).

Moreover, transgenic lines expressing the HDA:GFP transgene under the control of the 35S promoter of the *Cauliflower mosaic virus* were generated. Protoplasts from 3-week old leaves were isolated and observed for subcellular localization. However, the GFP signals elicited by these transgenic protoplasts were relatively weak (Figure S2). Nevertheless, HDA5, HDA8, and HDA14 were evidently distributed along the cytoplasmic area. On the other hand, HDA15 was confined exclusively inside the nucleus with GFP signals emanating in the nucleolus.

**Figure 3:**
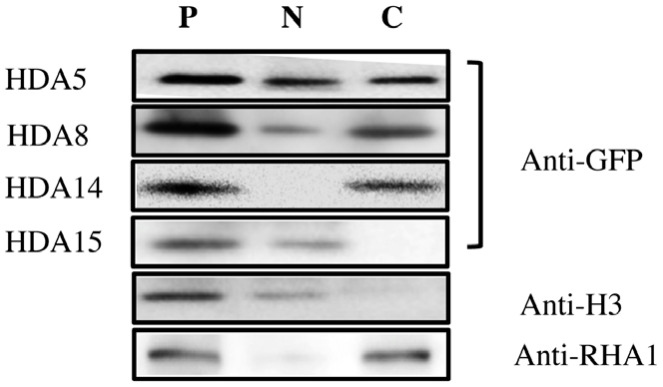
Cell fractionation and immunoblot detection HDA-GFP transfected protoplasts were separated into cytoplasmic and nuclear fractions then subjected to immunoblot analysis using anti-GFP antibody. Histone H3 and RHA1 were used as nuclear and cytoplasmic markers, respectively, on WT protoplasts. P protoplast extract, N nuclear fraction, C cytosolic fraction.

Based on these results, it is unclear whether HDA5, HDA8, and HDA14 are exclusively cytoplasmic or nuclear as well. To resolve this, cell fractionation and immunoblot detection was carried out. As illustrated in Figure 3, HDA5 and HDA8 were detected in both cytoplasma and nuclear fractions while HDA14 was exclusively cytoplasmic. On the other hand, HDA15 was restricted in the nucleus.

**Figure 4:**
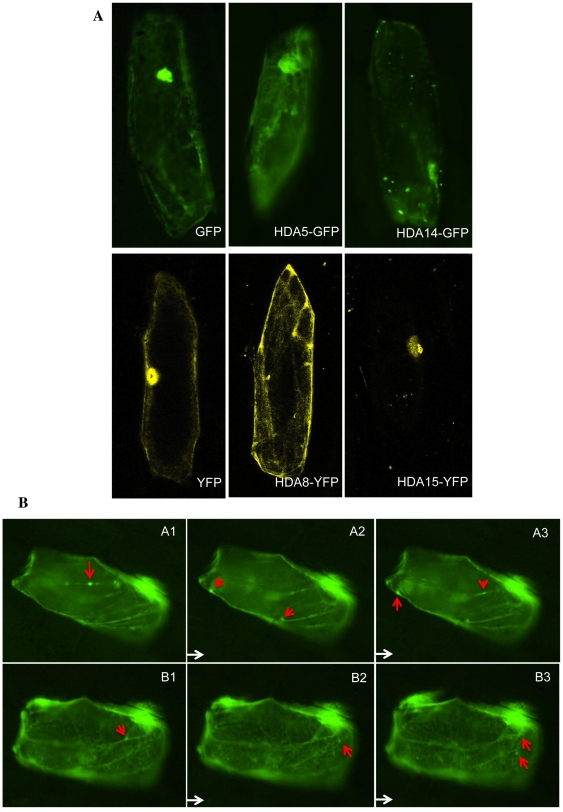
Particle bombardment in onion epidermal tissues. A. Onion tissues were transfected with HDA-GFP plasmids to visualize the pertinent localization of Class II HDAs. HDA5 and HDA8 both localize in the nucleus and cytoplasma while HDA14 cytoplasmic. Still, HDA15 remained nuclear. B. Onion tissues transfected with HDA5-GFP shows its localization in the cytoskeletal network with the dynamic movement of HDA5 spots (arrows) along these web-like structures. Pictures were taken at 2 sec intervals with the same onion cell.

To have a larger view of the HDA's organelle localization and dynamics, transient expression of HDA-YFP/GFP in onion epidermal tissues using particle bombardment was conducted. As shown in Figure 4A, HDA5 exhibited nuclear concentrations with well-defined enrichments along the cytoskeletal region. HDA8 localizes both in the nucleus and cytoplasm while HDA14 remains exclusively cytoplasmic with speckled distribution and refined localization within organelles. Still, HDA15 remained nuclear (Figure S3).

Although most of the GFP signals observed in all the Class II HDAs were static, we have discovered the dynamic movement of HDA5 along the cytoskeletal area (Figure 4B) suggesting a function potentially in tubulin deacetylation which may be similar to human HDAC6. This would explain the predominant HDA5 spots in transgenic protoplasts where the cytoskeletal area may be too thin or weak to exude GFP signals in comparison to the web-like signals in transient protoplasts, which strongly highlights the cytoskeleton obscuring the spots.

Based on these results, HDA5 and HDA8 were consistently observed in the cytoplasm with partial enrichments along the nuclear vicinity. On the other hand, HDA14 was distinctly localized in specific cytoplasmic organelle/s. In an attempt to identify specific organellar localization of these HDAs, subcellular markers were employed and co-transfected together with the HDA-YFP constructs in Arabidopsis PSB-D cell lines. These cells are devoid of chloroplasts to avoid ambiguous signals emitted from autofluorescence and reveal a clearer view of the localization of HDAs. Co-transfection was likewise employed in Col-0 protoplasts to investigate potential chloroplast distribution.

**Figure 5:**
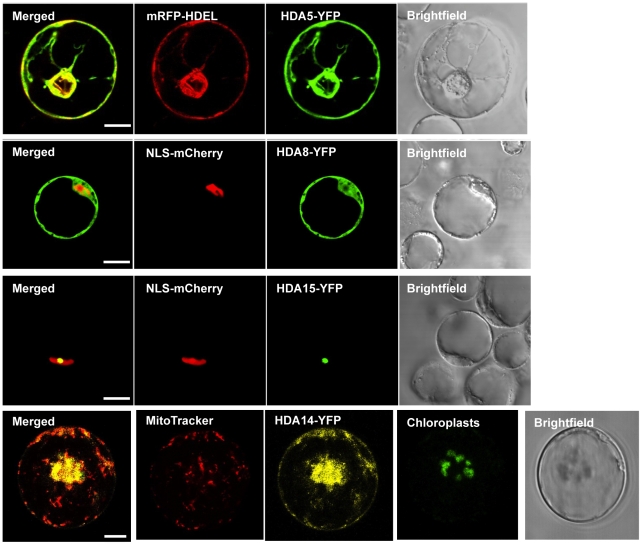
Organelle markers and Class II HDA localization. Specific organelle markers fused with RFP and mitoTracker stain were used to identify the subcellular localization of Class II HDAs. The ER marker, HDEL, overlaps with the localization of HDA5-GFP. Although HDA8 predominantly abounds in the cytoplasm, partial nuclear localization was observed at the surrounding areas of the nucleus using VirD2NLS as nuclear marker. Moreover, HDA15 concentrates in a small spot inside the nucleus, potentially nucleolus. On the other hand, HDA14-YFP was confirmed to localize in the chloroplasts and mitochondria using mitoTracker. Protoplasts derived from Arabidopsis PSB-D lines were used for PEG transfection in HDA5, HDA8, and HDA15 while protoplasts from Arabidopsis leaves were utilized for the HDA14-YFP localization. Scale bars were calibrated to 10 µm.

Cytoplasmic HDA5 shows striking co-localization with the cytoskeleton network. Since the endoplasmic reticulum is composed of an extensive network of cisternae held together by the cytoskeleton, an ER marker fused with mRFP was used. HDEL contains a targeting sequence with Lys-Asp-Glu-Leu residues found in the endoplasmic reticulum protein retention receptor1 first isolated in humans [60]. Overlay pictures bet YFP signals from HDA5 consistently matched the mRFP fluorescence from HDEL confirming the localization of HDA5 in the ER (Figure 5). It is probable HDA8 may generally be suspended in the cytosol as none of the organelle markers tested co-localized with its pertinent distribution. With the use of VirD2NLS as nuclear marker, HDA8 also occupies the peripheral areas of the nucleus in contrast to HDA15, which is strictly confined inside a small spot, potentially nucleolus. Moreover, HDA14-YFP was positively confirmed in the mitochondria with YFP signals co-localizing with the MitoTracker, a mitochondria specific stain. In addition, strong HDA14-YFP signals likewise overlapped with the chloroplasts.

### 3. Interaction of 14-3-3 with Class II HDAs

Human Class II HDACs have been subdivided as Class IIa, which is dependent on 14-3-3 for nuclear export and cytoplasmic retention, and Class IIb, which relies on its own NLS and NES for nuclear import and export. Although all the Class II HDAs in Arabidopsis contain three conserved Ser & Thr residues which are potential binding sites for 14-3-3, only HDA15 and HDA18 both contain nuclear localization (NLS) and nuclear export signals (NES) indicating their potential to be classified as Class IIb HDAs. Based on the neighbor joining phylogenetic tree generated from Class II HDAs in humans, *Drosophila*, *C. elegans*, and yeast, HDA5 and HDA18 are more closely related to HsHDA504, HsHDA505, HsHDA509, and HsHDA507, which have been well established as Class IIa HDACs. On the other hand, HDA15 is in the middle between Class IIa and Class IIb (HsHDA506 and HsHDA510) showing both potential to be classified as Class IIa and Class IIb [4].

**Figure 6:**
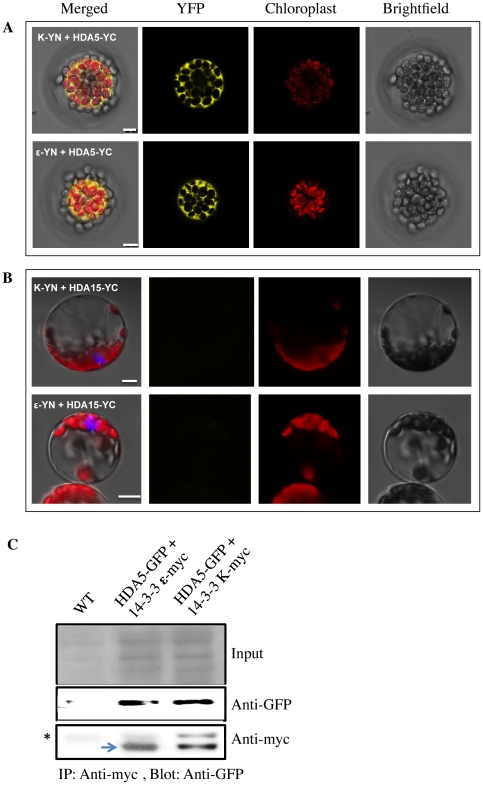
Interaction of HDA5 with 14-3-3. A. HDA5 exhibited positive interaction with 14-3-3 K and ε in the cytoplasm indicating that HDA5 requires these chaperones for its nuclear export and cytoplasmic retention considering that it does not contain any nuclear export signal within its amino acid sequence. B. On the contrary, HDA15 did not elicit any interaction with 14-3-3 K nor ε suggesting that it may rely on its own nuclear localization and export signals. C. CoIP results further confirm the positive association of HDA5 with 14-3-3 K and ε. HDA5-GFP was co-transfected into protoplasts with myc-tagged 14-3-3 K or 14-3-3 ε. HDA5 was detected using anti-GFP while 14-3-3 proteins were immunoprecipitated with anti-myc monoclonal antibody. Asterisk and arrowheads indicate non-specific bands and 14-3-3-myc, respectively.

To determine if these histone deacetylases can be subclassified as Class IIa or Class IIb, biflourescence complementation (BiFC) assay was undertaken to assess if HDA5 and HDA15 can interact with 14-3-3 κ and ε. 14-3-3 proteins are generally known to have high sequence homology with very low specificity to its target proteins. As shown in Figure 6, HDA5 exhibited positive interactions with both 14-3-3 κ and ε in the cytoplasm. Whether HDA5 was fused with the YFP amino end with 14-3-3 κ and 14-3-3 ε fused with the YFP carboxyl end or vice versa, consistent cytoplasmic interactions were observed. This interaction was further validated using coimmunoprecipitation where HDA-GFP was co-transfected with myc-tagged 14-3-3 κ or 14-3-3 ε (Figure 6C). Unfortunately, this association failed to interact in yeast-two- hybrid (data not shown) indicating the need for a kinase, which may be absent in yeast, to catalyze this interaction. Similar findings were also found in Class IIa human HDAC5, which positively binds with 14-3-3 *in vivo* but fails to associate in yeast suggesting its interaction to be largely dependent on Ca_2+_/calmodulin-dependent kinases [61]. These results suggest that HDA5 relies on 14-3-3 proteins for its nuclear export and cytoplasmic retention considering that it does not contain any NES. This classifies HDA5 as a Class IIa histone deacetylase. On the other hand, HDA15 failed to interact with either 14-3-3 κ or ε which may suggest its potential to rely on its own NES and NLS signals.

### 4. NLS and NES signals of HDA15 navigate its subcellular compartmentalization

**Figure 7:**
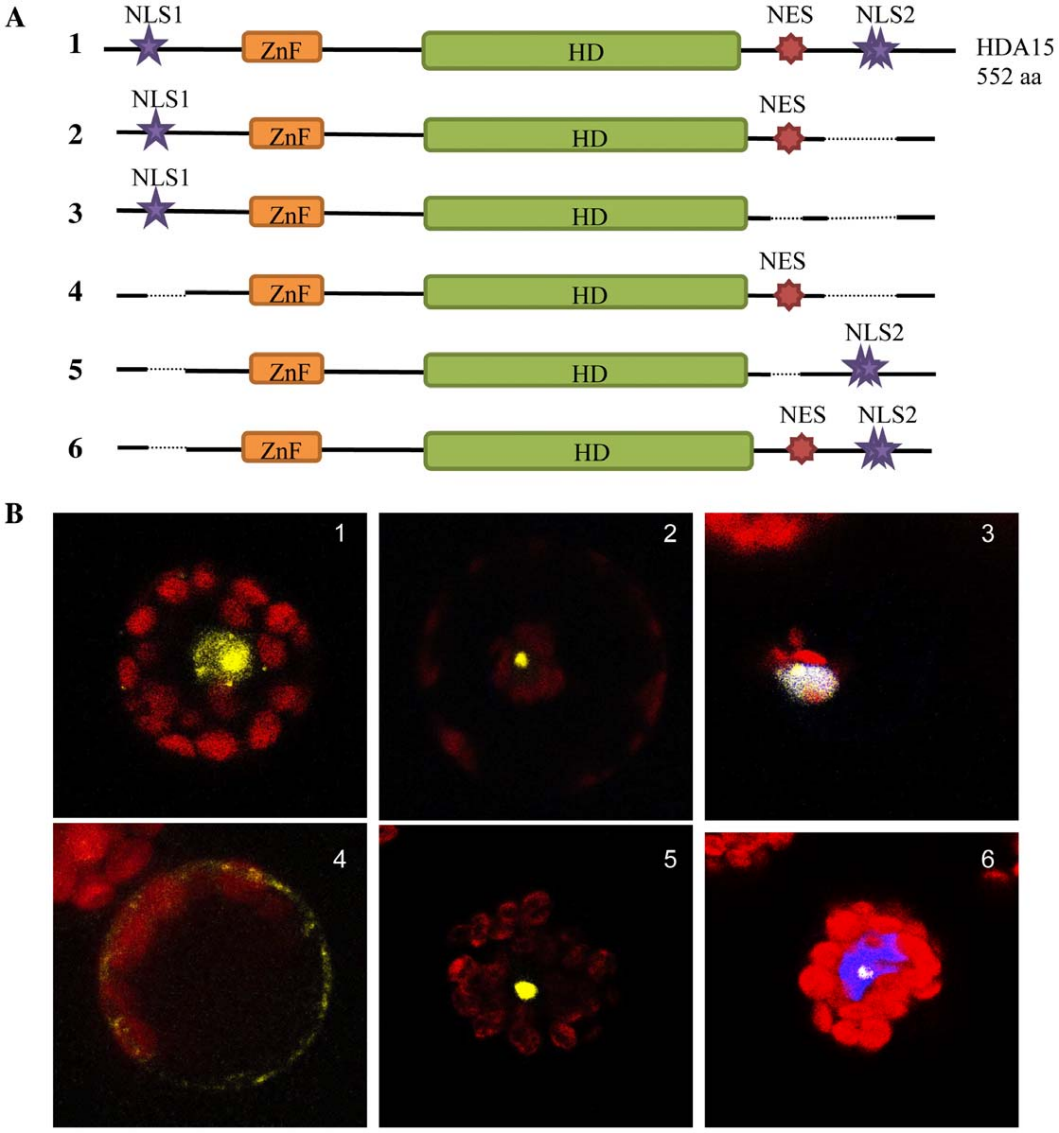
Nuclear localization and export signals of HDA15. A. Schematic representation of HDA15 is shown with its conserved histone deacetylase domain (HD), RanBP-type zinc finger (ZnF), nuclear localization signals (NLS), and nuclear export signal (NES). HDA15 contains three NLS signals, one classical par4 type NLS near the N-terminal and an overlapping bipartite NLS near the C-terminal end hereby jointly deleted as NLS2, and an NES stationed before the bipartite NLS near the carboxyl end. Numbers 1-6 illustrate the different truncated constructs of HDA15 where varying combinations of NLS and NES signals were deleted, shown here as dash lines. B. To determine if HDA15 depends on its NLS and NES for its subcellular localization, these predicted signals were truncated out of the HDA15-YFP and transiently expressed in protoplasts. Both NLS1 and NLS2 functionally direct the nuclear localization of HDA15 and remains active even in the absence of the other. However, the deletion of both NLS renders HDA15 cytoplasmic indicating the functionality of its NES. This suggests that HDA15 can translocate in and out of the nucleus and potentially undergo nucleocytoplasmic shuttling.

HDA15 contains three NLS signals, one classical par4 type NLS near the N terminal end and two overlapping bipartite NLS near the C terminal end (Figure 7A). The predicted NES was stationed before the second and third NLS near the carboxyl end. Since the second and third NLS were overlapping, they were jointly omitted as NLS2. To test whether these NLS and NES functionally navigate the localization of HDA15, truncated constructs were generated deleting these predicted signals from HDA15-YFP. As shown in Figure 7B, the removal of either NLS1 or NLS2 still renders HDA15 nuclear indicating that both NLS are functional. In the absence of the other, one can still direct the nuclear localization of HDA15. However, the deletion of both NLS signals culminates into its nuclear export signifying the functionality of its NES. Taken together, these results demonstrate that HDA15 can translocate in and out of the nucleus whereby its nuclear localization and export signals navigate its subcellular compartmentalization. This classifies HDA15 as a Class IIb histone deacetylase.

### 5. Nuclear localization of HDA15 is strongly expressed in stems and leaves

**Figure 8:**
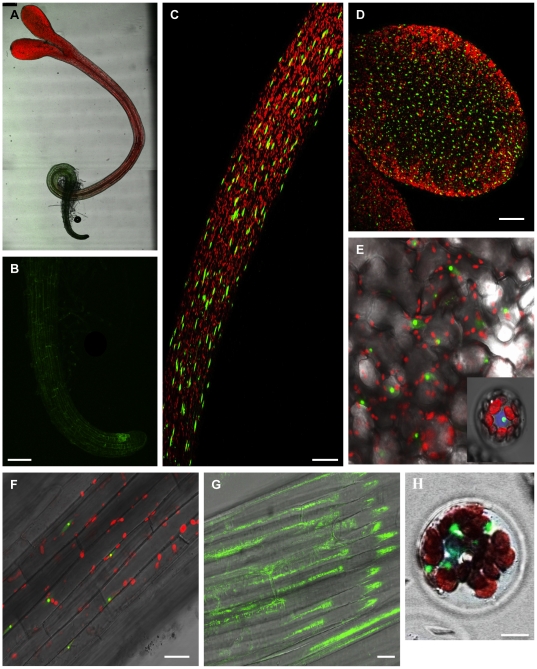
Whole plant localization of HDA15-GFP in 4-day old seedlings. Four day old de-etiolated seedlings of HDA15-GFP were observed for subcellular localization with the projection confocal image of the entire seedling (A), root tip (B), hypocotyl (C), and cotyledon (D). A magnified view of the leaf (E) and hypocotyl cells (F) of long day grown seedlings reveal nuclear concentrations of HDA15-GFP. On the contrary, those grown in total darkness for 4 consecutive days exhibited cytoplasmic localization of HDA15-GFP (G). In concurrence with this, transgenic protoplasts revealed nuclear confinements of HDA15-GFP upon white light treatment (E) similar to transfected protoplasts (inset E). However, 3-hour dark treated transfected protoplasts after 18 h of white light incubation resulted to its cytoplasmic translocation (H). VirD2NLS was co-transfected as nuclear marker (blue). Red color indicates autofluorescence emitted by chloroplasts. Scale bars for A to D is 100 µm, F & G was set at 25 µm, and H at 10 µm.

Although HDA15 was clearly observed to be nuclear in both transgenic and transfected protoplasts, its mRNA expression patterns in the different vegetative organs varied with strong preference for the stems and leaves. To confirm these findings, HDA15-GFP transgenic lines were grown on 1/2 MS media to assess differences in its signal and localization. As demonstrated in Figure 8, whole plant localization of HDA15-GFP in four day old de-etiolated seedlings exhibited nuclear confinements of HDA15 in the hypocotyl and leaf protoplasts. However, weak GFP signals were observed in the root (Figure 8B). Although relatively weak, the strongest signal in the root appears to concentrate at the quiescent center. Light exposed organs, namely hypocotyl and cotyledons (Figure 8C–F), exhibited very strong nuclear GFP signals. On the other hand, etiolated seedlings grown in the dark for four days elicited predominantly cytoplasmic concentrations in the hypocotyl (Figure 8G). These results were further confirmed using transient expression of HDA15-GFP in protoplasts incubated under white light for 18 h displaying nuclear concentrations of HDA15 (Figure 8E inset) similar to transgenic protoplasts. Separating the white light spectrum into far red, red, and blue light as light treatment for 3 h after 18 h white light incubation similarly resulted into its nuclear localization suggesting that light quality does not affect its localization (Figure S5). On the contrary, transfected protoplasts treated in complete darkness for 3 h after 18 h of white light incubation generated cytoplasmic localization of HDA15 (Figure 8H). This indicates that the nuclear localization of HDA15 is signaled by light and is not influenced by any specific wavelength.

### 6. Light drives the nucleocytoplasmic shuttling of HDA15

**Figure 9:**
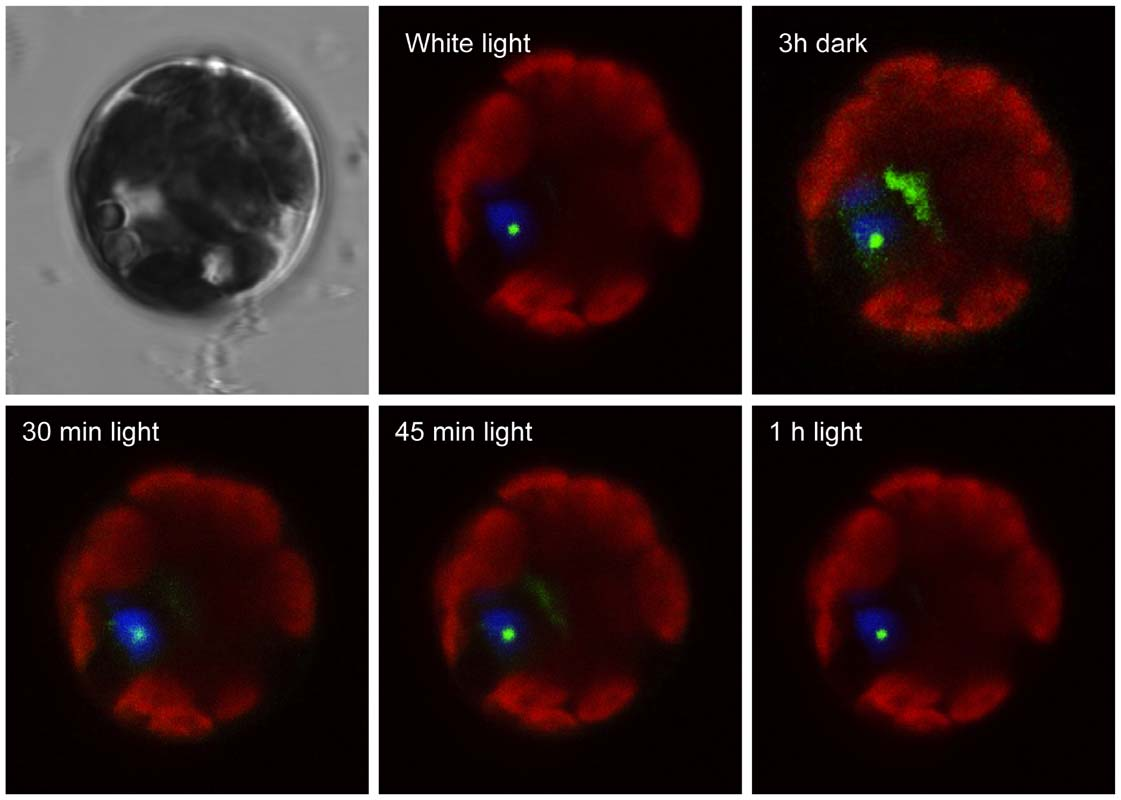
Nucleocytoplasmic shuttling of HDA15. To illustrate if HDA15-GFP undergoes nucleocytoplasmic shuttling, transfected protoplasts were incubated under white light overnight then covered with foil for 3 hours then re-exposed to white light. HDA15-GFP transfected protoplasts exhibited nuclear localization after 18 h of white light incubation. Further dark treatment for 3 h elicited partial cytoplasmic translocation of HDA15-GFP. Re-exposition of these protoplasts to white light after one hour lead to its complete nuclear import clearly demonstrating that light drives the nucleocytoplasmic shuttling of HDA15. VirD2NLS was co-transfected as nuclear marker (blue). Scale bars were calibrated to 10 µm.

The nuclear and cytoplasmic localizations of HDA15 in the presence and absence of light, respectively, unveil its strong potential to undergo nucleocytoplasmic shuttling. This functional regulatory mechanism may control its activation and inactivation as a histone deacetylase just like its corresponding orthologues in humans and other eukaryotes. To determine if plant Class II HDAs, HDA15 in particular, can also display nucleocytoplasmic shuttling, HDA15-GFP transfected protoplasts were incubated under white light overnight then covered with two layers of foil for three hours then re-exposed to white light. As shown in Figure 9, HDA15-GFP transfected protoplasts exhibited nuclear localization after 18 h of white light incubation. Further dark treatment elicited cytoplasmic translocation of HDA15. Re-exposition of these protoplasts to white light after one hour lead to its complete nuclear import clearly demonstrating that light drives the nucleocytoplasmic shuttling of HDA15.

## Discussion

### Class II HDAs display minimal developmental specificity and are strongly expressed in stems

Unlike human Class II HDACs which exhibit strict developmental specificity, Arabidopsis Class II HDAs display minimal developmental specificity and are ubiquitously expressed in all tissues with stronger signals in stems. This affirms our previous bioinformatics data where Class II HDAs have been predicted to be prominently expressed in stems [4]. HDA5 and HDA8 may display abundant transcript levels in stems, leaves, and mature seeds, however, they may only be active at certain life stages of the plant. On the contrary, HDA14 and HDA15 are homogenously expressed all throughout its developmental stages but mostly in stems. In addition, HDA18 abounds in the roots and stems but inactive during young flower stage. Prior studies by Xu *et al.* [19] have implicated the role of HDA18 on root epidermal patterning such that reduced trichostatin A (TSA) treatment deregulates the expression of key patterning genes GLABRA (GL2), CAPRICE (CPC), and WEREWOLF (WER). Considering the same genes are relatively active in trichome development and patterning in leaves, the involvement of HDA18 on epidermal patterning maybe exclusive on the roots as its expression is kept at a minimum in the leaves. The strong expression profile of Class II HDAs in stem stirs baffling questions with the possibility of the involvement of this entire class of histone deacetylases in hypocotyl or stem development.

### Cytoplasmic localization of HDA5, HDA8, and HDA14 entails their potential for nuclear transport and versatility as deacetylases of non-histone proteins

The translocation of human Class II HDACs into the cytoplasm is generally considered as a functional regulatory mechanism, which inactivates its catalytic activity as a histone deacetylase via subcellular compartmentalization. However, they can also target cytosolic substrates especially when bound to specific organelles. Plant Class II HDAs may exhibit similar functional regulation as well as versatile roles since all the Class II HDAs were observed to be cytoplasmic. HDA5 prominently localizes in the cytoplasm, lining the nucleus, in distinct web like structure resembling the cytoskeleton, which was confirmed by the use of HDEL, an endoplasmic reticulum marker. The ER is composed of an extensive network of cisternae held together by the cytoskeleton. Studies by Mi and Puglielli [62] have implicated the presence of HATs and HDACs in the ER and Golgi describing the reversible acetylation of BACE1, a protease responsible for amyloid precursor protein cleavage, and LDL receptor, respectively. Although the HAT responsible for this occurrence has been determined, the identity of the HDAC still remains to be discovered. It is probable that the human orthologue of HDA5 could catalyze the deacetylation of these proteins. Furthermore, the dynamic movement of HDA5 along the cytoskeletal network in onion tissues implicates its potential function in tubulin deacetylation which may be similar to human HDAC6. Phylogenetic analysis conducted by Gregoretti *et al.* [63] proposes HDA5 to be the plant orthologue of mammalian HDAC6 which deacetylates alpha- tubulin similarly conserved in all eukaryotes. But unlike human HDAC6, which is exclusively cytoplasmic and depends on its own NLS and NES, HDA5 localizes both cytoplasm and nucleus and positively binds with 14-3-3 potentially for its nuclear export and cytoplasmic retention since it does not have any NES. Similar to the human Class IIa HDAC5, the inability of HDA5 to bind with 14-3-3 K and E indicates that specific kinases which are absent in yeast, are required to catalyze the phosphorylation of 14-3-3 for its binding to HDA5. In humans and other model organisms, five kinase groups have been shown to phosphorylate Class IIa HDACs. These include Ca_2+_/calmodulin-dependent kinases [61], [64], salt-inducible kinases [65], [66], protein kinase D [67]–[69], microtubule affinity-regulating kinases [70], [71], and checkpoint kinase-1 [72]. It is highly likely that microtubule affinity-regulating kinases may be involved in phosphorylating 14-3-3 binding sites with HDA5 considering that it localizes in the cytoskeletal region. Nevertheless, more studies are needed to elucidate further the mechanisms governing HDA5 and 14-3-3 interaction as well as the exact substrates and function of HDA5 in plants.

HDA8 prominently occupies the cytoplasm as well as peripheral areas of the nucleus. However, subcellular markers including ER, golgi, trans-golgi network, prevacuolar compartment, and mitochondria staining failed to overlap with its pertinent localization suggesting HDA8 to be suspended in the cytoplasma. Compared to all the Class II HDAs in Arabidopsis, it is the sole histone deacetylase devoid of an NLS but with a NES imprinted along the histone deacetylase region, which is similar to the conserved HDA domain of human Class IIa HDACs. In the absence of an NLS, HDA8 may need to associate with other proteins that would chaperone its translocation to undergo nuclear import. Although its conserved histone deacetylase domain is homologous to Class II HDAs compared to the other members of the RPD3/HDA1-like superfamily, it is more probable that its function is stationed in the cytoplasm targeting the deacetylation of non-histone proteins. The cytoplasmic localization of Class II HDAs may not necessarily entail inactivity but its enzymatic target clients may encompass beyond nuclear histones expanding its substrate repertoire.

The speckled distribution of HDA14 was confirmed in the mitochondria and chloroplasts similar to its rice orthologue, OsHDAC10 [22]. Based on iPSORT and SignalP prediction programs, HDA14 contains a mitochondrial and chloroplast transit peptide encoded at amino acids 4 to 11, yet, still has the potential to translocate into the nucleus considering that a classical type, pat7 NLS is embedded in its sequence near the carboxyl end. Its similarity to HDA5, HDA15, and HDA18 could be this nuclear importation given the proper signals to pool it towards the nucleus.

It is worth noting though that HDA8 and HDA14 are uniquely conserved only in plants. The potential function of HDA8 in the cytoplasm and HDA14 in the mitochondria and chloroplasts may involve regulatory mechanisms in energy homeostasis similar with the human NAD-dependent sirtuin family [73]. HsSirt3, in particular, translocates into the mitochondria upon stress [74]. In this case, however, it could be the reverse such that HDA14 will only translocate into the nucleus given the proper environmental signals. Studies by Wallace and Fan have elaborated on the dynamic interplay between bioenergetics and epigenetics linking human epigenetic disorders with mitochondrial dysfunction [75], [76]. In the context of plants, however, energy regulatory dynamics is complicated by light harvesting, inorganic chemosynthesis, to carbon fixation. Moreover, its static nature exposes plants to a wider scope of stress implicating greater magnitude of epigenomic changes thus its survival tactics are more intricately complex than mobile organisms. Histone acetylation, in particular, has been associated in regulating gene expression in developmental transitions [7], [5], varying environmental signals such as light [77] and low temperature [78], and stress hormone signals [79].

In addition, the cytoplasmic localization of HDA8 and HDA14 in plants may entail crucial roles in deacetylating proteins essential in photosynthesis and other cytoplasmic metabolic pathways. Recent studies by Finkemier *et al.* [20] and Wu *et al.* [21] have pioneered a systematic study on non-histone proteins targeted for Lys acetylation in Arabidopsis. Based on their results, a significant proportion of Lys- acetylated proteins are involved in photosynthesis, TCA cycle, glycolysis, structural proteins, cell signaling, and plant stress responses. Of particular interest is the deacetylation of multiple Lys groups in the large subunit of rubisco using the human Sirt3, which induced a 40% increase in its maximum catalytic activity. Assuming HDA8 and HDA14 directly deacetylate any of these photosynthetic proteins and metabolic enzymes, this would justify its evolutionary divergence from heterotrophs intricately accessorizing itself with a more efficient energy regulatory response to balance the dynamic interplay between the environment, chromatin modification, and bioenergetics.

### Light drives the nucleocytoplasmic shuttling of HDA15

The nuclear importation of HDA15 in the presence of light may implicate its functional activity within the light signaling pathway. More so, its expression is mostly abundant in the stems and leaves all throughout the developmental stages of the plant. Thus, we cannot exclude the possibility that HDA15 may perform active roles in photomorphogenesis.

Moreover, the localization of HDA15 appears to be mostly restricted inside a confined spot inside the nucleus, possibly nucleolus suggesting a function in deacetylating nucleolar core histones. This study have paved the baseline evidence that plant Class II HDAs, HDA15 in particular, can undergo nucleocytoplasmic shuttling with light signaling its complete nuclear transport. However, it is puzzling to note why and how HDA15 specifically drives into the center since differences in light exposure or wavelength do not influence its nuclear distribution. Furthermore, it is yet to be determined whether its cytoplasmic export functionally regulates its inactivity or remains active targeting the deacetylation of cytoplasmic proteins.

## Materials and Methods

### Plant Material & Growth Conditions

*Arabidopsis thaliana* ecotype Col-0 was used as wild type and genetic background for all transgenic lines. Seeds were cold-treated for 2–3 days, sown on 1/2 strength Murashige & Skoog media, and then transferred to a growth chamber for germination at 20–24°C under long day conditions (16 h light/8 h dark cycle). Seedlings were further grown either in medium or soil pots. Prior to sample collection for mRNA analysis, developmental stages of the plant were observed and compared with the timeline indicated in Genevestigator using 20 replicates in each of the 3 trials.

### RNA Extraction & RT-PCR

Gene expression was assessed using semi-quantitative RT-PCR. Total RNA was extracted from plant samples weighing 0.25 to 0.3 g using TRIZOL reagent (Invitrogen). Oligo(dT) primed reverse transcription of first strand cDNA synthesis was carried out with 7 µg total RNA using SuperScript_TM_ III (Invitrogen). Equal volumes of each first strand reaction were amplified with gene-specific primer pairs. Thermocycling conditions were 94°C for 4 mins followed by 30 cycles of 94°C for 30 s, 55–60°C for 30 s, and 74°C for 1–2 min. Primer sequences are available upon request.

### HDA-YFP/GFP fusion constructs

Coding sequences of Class II histone deacetylase were amplified by PCR from expressed sequence tags (RIKEN) and subsequently cloned into the entry vector, pENTR/SD/D-TOPO or PCR8/GW/TOPO, with spectromycin as bacterial marker. An LR clonase enzyme mix (Invitrogen) was used to transfer the insert from entry clones to its destination vectors, p2FGW7 with the GFP tag positioned at the N-terminal of the insert, and p2YGW7 which contains the YFP tag at the C-terminal of the gene. Both destination vectors are high copy vectors driven by a double 35S cauliflower mosaic virus promoter with ampicillin as bacterial marker. Purified plasmids were then analyzed and sequenced to confirm successful fusion constructs.

### Plant Transformation

Class II HDAs in pENTR vectors were used for recombination to a binary vector, pK7WGF2, which contains a 35S CaMV promoter and a GFP tag at the carboxyl end of the insert. Spectromycin was used as bacterial marker. Purified plasmids were sequenced to confirm successful insertion and integrated into the Arabidopsis genome by *Agrobacterium tumefaciens*-mediated floral dipping method [80], [81]. Seeds from transformed plants were germinated on 1/2 strength MS media with kanamycin for plant selection. Seven-day-old kanamycin resistant seedlings were then screened under fluorescence microscope for GFP signals then transplanted into soil pots for 3 weeks for seed production. T2 and T3 seedlings were used for the studies described herein.

### Protoplast Isolation and Transient Expression

Leaves of 3-week old T2 transgenic lines were used to determine subcellular localization in protoplasts. Transient expression assays were subscribed from the methods of Yoo *et al.* [82] with some modifications. Mesophyll protoplasts were isolated from 3-week old Col-0 plants and Arabidopsis PSB-D cell lines [83]. Twenty µg of HDA-YFP/GFP fusion plasmid, VirD2-NLS as nuclear marker [84] and organelle markers [85] were co-transfected into 4×10^4^ protoplasts using polyethylene glycol (PEG) solution (0.4 g/ml PEG 4000, 0.8 M mannitol, 125 mM CaCl_2_), incubated for 5–15 min at room temperature, washed and resuspended in W5 solution (154 mM NaCl, 125 mM CaCl_2_, 5 mM KCl, 2 mM MES at pH5.7), then incubated under white light for 16–24 h prior to imaging using Leica SP5 confocal microscope. For mitochondria staining, protoplasts were immersed in 0.2 µM MitoTracker Orange CMTMRos (Invitrogen, M7510) for 15 mins then mounted on slide with W5 solution.

For the localization of HDA15 in different light treatments, transfected protoplasts were incubated under white light for 18 h then transferred to E30LEDL3 growth chambers (Percival Scientific) with far red, red, and blue light-emitting diode sources for 3 h. Low light intensities used as treatment were measured at 2.77 µmol m^−2^ s^−1^ (FR), 1.77 µmol m^−2^ s^−1^(R), and 3.84 µmol m^−2^ s^−1^(B). For transgenic plants, seeds were grown in 1/2 MS media inside a growth chamber with 16 h light–8 h night cycle for white light treated seedlings while dark treated seeds were wrapped in foil and grown inside a dark growth chamber for 3–4 days.

### Transient Expression in Onion Epidermal Tissues

Two µg of HDA-GFP fusion plasmids were coated on 20 µl of 50 mg ml^−1^ gold particles with 2.5 M CaCl_2_ and 0.1 M spermidine then mixed rigorously using a vortex for 2 min. Plasmid-coated particles were dehydrated with 75% and 95% ethanol prior to bombardment. Single layer epidermal sheaths peeled from onion bulbs were placed on ½ MS plates then subjected to particle bombardment using the standard procedure provided by the manufacturer. Plasmid-coated gold particles were accelerated with a helium burst at 1100 psi in a PDS-1000/He instrument (BioRad). Plates containing transfected onion tissues were wrapped in foil and kept in the dark overnight (16–20 h) at room temp.

### Cell Fractionation and Immunodetection

Nuclear and cytosolic fractions in protoplasts expressing HDA-GFP proteins were separated following the methods previously described by Ryu *et al.* [86] and Yanagisawa *et al.* [87]. Immunoblotting was carried out using 15–20 µg of proteins from transfected protoplasts, nuclear and cytosolic fractions were resolved by 10% SDS-PAGE, and detected with horseradish peroxidase-conjugated anti-GFP (Clontech), anti-RHA1 (Upstate), and anti-histone H3 (Upstate) antibodies [88].

### Protein-Protein Interaction Assays

For the biflourescence complementation (BiFC) assay, coding sequences of HDA5, HDA15, and 14-3-3 cloned into PCR8/GW/TOPO were used for recombination into the destination vectors, pEarleyGate201-N-YFP and pEarleyGate202-C-YFP [89] using LR recombination mix. Kanamycin was used for bacterial selection. Purified plasmids were then analyzed for DNA sequencing for confirmation and further used for PEG transfection and imaging. Negative controls with empty vectors and test constructs and positive controls with BZR1 and 14-3-3s were initially tested to assess the efficiency of the BiFC assay (Figure S4). Coimmunoprecipitation (CoIP) was carried out using the methods of Ryu *et al.* [86] with some modifications. HDA-GFP plasmids with co-transfected with myc-tagged 14-3-3 K or E and then incubated for 6–8 h for protein expression. Total protein was extracted from transfected protoplasts using immunoprecipitation buffer (50 mM Tris-HCl, pH 7.5, 75 mM NaCl, 5 uM EDTA, 1 mM DTT, protease inhibitor cocktail [Roche Applied Science], and 1% TritonX-100). Protein complex was precipitated with monoclonal anti-c-myc antibody (Cell Signaling) and protein A/G plus-agarose beads (Calbiochem). Precipitated proteins were detected with horseradish peroxidase-conjugated anti-GFP antibody (Clontech). For yeast-two-hybrid assay, yeast strain AH109 was transformed with pGBKT7 vector expressing HDA5 and pGADT7 vector expressing 14-3-3 K or E. Transformed cells were grown on synthetic media without Leu, Trp, and His containing 3 mM 3-aminotriazole or medium lacking Leu and Trp.

## Supporting Information

Figure S1 **Protoplast transient expression using GFP-HDA fusion constructs.** Subcellular localization of Class II HDAs was determined via protoplast PEG transfection using GFP-HDA fusion constructs. HDA5, HDA8, and HDA14 were cytoplasmic while HDA15 was restricted inside the nucleus. VirD2NLS fused with mCherry was used as a nuclear marker. Scale bars were calibrated to 10 µm.
(TIF)

Figure S2 **Subcellular localization in transgenic protoplasts.** Protoplasts from transgenic lines of Class II HDAs were also used to determine their corresponding subcellular localization. Although GFP signals were relatively weak, HDA5, HDA8, and HDA14 were found to abound in the cytoplasm while HDA15 emanated strong nuclear signals.
(TIF)

Figure S3 **Particle bombardment in onion tissues.** Overlay pictures reveal the nuclear and cytoplasmic localization of HDA8-YFP and the nuclear concentration of HDA15.
(TIF)

Figure S4 **BiFC negative and positive controls.** Empty vectors and YN/YC constructs were tested as negative controls. BZR1 was used as a positive control for the 14-3-3 kappa and epsilon interactions.
(TIF)

Figure S5 **Nucleolar localization of HDA15-YFP in different light treatments.** Transfected protoplasts were incubated under white light for 18 h then transferred to far red, red, and blue light treatments for 3 h at low light intensities (FR 2.77 µmol m^−2^ s^−1^, R 1.77 µmol m^−2^ s^−1^. B 3.84 µmol m^−2^ s^−1^). Similar with white light treated protoplasts, HDA15-YFP was restricted in a small spot inside the nucleus, potentially nucleolus. VirD2NLS-mCherry was co-transfected as a nuclear marker (blue). Scale bars were calibrated to 10 µm.
(TIF)

## References

[1] Pandey R, Muller A, Napoli C, Selinger D, Pikaard C (2002). Analysis of histone acetyltransferases and histone deacetylase families in *Arabidopsis thaliana* suggests functional diversification of chromatin modifications among multicellular eukaryotes.. Nuc Acid Res.

[2] Wu K, Tian L, Malik K, Brown D, Miki B (2000). Functional analysis of HD2 histone deacetylase homologues in *Arabidopsis thaliana*.. Plant J.

[3] Hollender C, Lui Z (2008). Histone deacetylase genes in Arabidopsis development.. J Integ Plant Biol.

[4] Alinsug MV, Yu CW, Wu K (2009). Phylogenetic analysis, subcellular localization, and expression patterns of RPD3/HDA1 family histone deacetylases in plants.. BMC Plant Biology.

[5] Tian L, Chen ZL (2001). Blocking histone deacetylation in Arabidopsis induces pleitropic effects on plant gene regulation and development.. Proc Natl Acad Sci USA.

[6] Tian L, Wang JJ, Fong MP, Chen M, Cao H (2003). Genetic control of developmental changes induced by disruption of Arabidopsis histone deacetylase 1 (AtHD1) expression.. Genetics.

[7] Tian L, Fong MP, Wang JJ, Wei NE, Jiang H (2005). Reversible histone acetylation and deacetylation mediate genome-wide, promoter-dependent and locus- specific changes in gene expression during plant development.. Genetics.

[8] Benhamed M, Bertrand C, Servet C, Zhou DX (2006). Arabidopsis GCN5, HD1, and TAF1/HAF2 interact to regulate histone acetylation required for light-responsive gene expression.. Plant Cell.

[9] Long JA, Ohno C, Smith ZR, Meyerowitz EM (2002). TOPLESS regulates apical embryonic fate in Arabidopsis.. Science.

[10] Tanaka M, Kikuchi A, Kamada H (2008). The Arabidopsis histone deacetylases HDA6 and HDA19 contribute to the repression of embryonic properties after germination.. Plant Physiol.

[11] Kim KC, Lai Z, Fan B, Chen Z (2008). Arabidopsis WRKY38 and WRKY62 transcription factors interact with histone deacetylase 19 in basal defense.. Plant Cell.

[12] Yu CW, Liu X, Luo M, Chen C, Lin X (2011). HDA6 interacts with FLD and regulates flowering in Arabidopsis.. Plant Physiol.

[13] To TK, Nakaminami K, Kim JM, Morosawa T, Ishida J (2011). Arabidopsis HDA6 is required for freezing tolerance.. Biochem Biophys Res Commun.

[14] Chen LT, Luo M, Wang YY, Wu K (2010). Involvement of Arabidopsis histone deacetylase HDA6 in ABA and salt stress response.. J Exp Bot.

[15] Wu K, Zhang L, Zhou C, Yu CW, Chaikam V (2008). HDA6 is required for jasmonate response, senescence and flowering in Arabidopsis.. J Exp Bot.

[16] Earley K, Lawrence RJ, Pontes O, Reuther R, Enciso AJ (2006). Erasure of histone acetylation by Arabidopsis HDA6 mediates large-scale gene silencing in nucleolar dominance.. Genes Dev.

[17] Chen LT, Wu K (2010). Role of histone deacetylases HDA6 and HDA19 in ABA and abiotic stress response.. Plant Signal Behav.

[18] Zhou C, Zhang L, Duan J, Miki B, Wu K (2005). HISTONE DEACETYLASE19 is involved in jasmonic acid and ethylene signaling of pathogen response in Arabidopsis.. Plant Cell.

[19] Xu CR, Liu C, Wang YL, Li LC, Chen WQ (2005). Histone acetylation affects expression of cellular patterning genes in the Arabidopsis root epidermis.. Proc Natl Acad Sci USA.

[20] Finkemeier I, Laxa M, Miguet L, Howden AJM, Sweetlove LJ (2011). Proteins of diverse function and subcellular localization are lysine acetylated in Arabidopsis.. Plant Physiol.

[21] Wu X, Oh MH, Schwarz EM, Larue CT, Sivaguru M (2011). Lysine acetylation is a widespread protein modification for diverse proteins in Arabidopsis.. Plant Physiol.

[22] Chung PJ, Kim YS, Park SH, Nam BH, Kim JK (2009). Subcellular localization of rice histone deacetylases in organelles.. FEBS Letters.

[23] Yang XJ, Gregoire S (2005). Class II histone deacetylases: from sequence to function, regulation, and clinical implication.. Mol Cell Biol.

[24] Verdin E, Dequiedt F, Kasler HG (2003). Class II histone deacetylases: versatile regulators.. Trends Genet.

[25] Nishino T, Miyazaki M, Hoshino H, Miwa Y, Horinouchi S (2008). 14- 3-3 regulates the nuclear import of class IIa histone deacetylases.. Biochem Biophys Res Comm.

[26] Wang AH, Yang X (2001). Histone deacetylase 4 possesses intrinsic nuclear import and export signals.. Mol Cell Biol.

[27] Bakin RE, Jung MO (2004). Cytoplasmic sequestration of HDAC7 from mitochondrial and nuclear compartments upon initation of apoptosis.. J Biol Chem.

[28] Gregoire S, Yang XJ (2005). Association with Class IIa histone deacetylases upregulates sumoylation of MEF2 transcription factors.. Mol Cell Biol.

[29] Hubbert C, Guardiola A, Shao R (2002). HDAC6 is a microtubule-associated deacetylase.. Nature.

[30] Matsuyama A, Shimazu T, Sumida Y (2002). In vivo destabilization of dynamic microtubules by HDAC6-mediated deacetylation.. EMBO J.

[31] Zhang Y, Li N, Caron C (2003). HDAC6 interacts with and deacetylates tubulin and microtubules in vivo.. EMBO J.

[32] Zhang X, Yuan Z, Zhang Y (2007). HDAC6 modulates cell motility by altering the acetylation level of cortactin.. Mol Cell.

[33] Bali P, Pranpat M, Bradner J (2005). Inhibition of histone deacetylase 6 acetylates and disrupts the chaperone function of heat shock protein 90: a novel basis for antileukemia activity of histone deacetylase inhibitors.. J of Biol Chem.

[34] Aoyagi S, Archer TK (2005). Modulating molecular chaperone Hsp90 functions through reversible acetylation.. Trends Cell Biol.

[35] Scroggins BT, Robzyk K, Wang D (2007). An acetylation site in the middle domain of Hsp90 regulates chaperone function.. Mol Cell.

[36] Kovacs JJ, Murphy PJM, Gailliard S (2005). HDAC6 regulates Hsp90 acetylation and chaperone-dependent activation of glucocorticoid receptor.. Molecular Cell.

[37] Li Y, Zhang H, Polakiewicz RD, Yao TP, Comb MJ (2008). HDAC6 is required for epidermal growth factor-induced B-catenin nuclear localization.. J of Biol Chem.

[38] Parmigiani RB, Xu WS, Venta-Perez G (2008). HDAC6 is a specific deacetylase of peroxiredoxins and is involved in redox regulation.. Proc Natl Acad Sci USA.

[39] McKinsey TA, Zhang CL, Olsen EN (2000). Activation of myocyte enhancer factor-2 transcription factor by calcium/calmodulin-dependent protein kinase- stimulated binding of 14-3-3 to histone deacetylase 5.. Proc Natl Acad Sci USA.

[40] Oecking C, Jaspert N (2009). Plant 14-3-3 proteins catch up with their mammalian orthologs.. Curr Opin Plant Biol.

[41] Sehnke PC, Rosenquist M, Alsterfjord M, DeLille J, Sommarin M, Larsson C, Ferl RJ (2002). Evolution and isoform specificity of plant 14-3-3 proteins.. Plant Mol Biol.

[42] Sehnke PC, DeLille JM, Ferl RJ (2002). Consummating signal transduction: the role of 14-3-3 proteins in the completion of signal-induced transitions in protein activity.. Plant Cell.

[43] Roberts MR (2003). 14-3-3 proteins find new partners in plant cell signaling.. Trends Plant Sci.

[44] Ferl RJ (2004). 14-3-3 proteins: regulation of signal-induced events.. Physiol Plant.

[45] Paul AL, Liu L, McClung S, Laughner B, Chen S (2009). Comparative interactomics: analysis of Arabidopsis 14-3-3 complexes reveals highly conserved 14- 3-3 interactions between humans and plants.. J Proteome Res.

[46] Chang IF, Curran A, Woolsey R, Quilici D, Cushman JC (2009). Proteomic profiling of tandem affinity purified 14-3-3 protein complexes in *Arabidopsis thaliana*.. Proteomics.

[47] Huber SC, MacKintosh C, Kaiser WM (2002). Metabolic enzymes as targets for 14- 3-3 proteins.. Plant Mol Biol.

[48] Bunney TD, van den Wijngaard PW, de Boer AH (2002). 14-3-3 protein regulation of proton pumps and ion channels.. Plant Mol Biol.

[49] Igarashi D, Ishida S, Fukazawa J, Takahashi Y (2001). 14-3-3 proteins regulate intracellular localization of the bZIP transcriptional activator RSG.. Plant Cell.

[50] Ishida S, Fukazawa J, Yuasa T, Takahashi Y (2004). Involvement of 14-3- 3 signaling protein binding in the functional regulation of the transcriptional activator REPRESSION OF SHOOT GROWTH by gibberellins.. Plant Cell.

[51] Schoonheim PJ, Sinnige MP, Casaretto JA, Veiga H, Bunney TD (2007). 14-3-3 adaptor proteins are intermediates in ABA signal transduction during barley seed germination.. Plant J.

[52] Lopez-Molina L, Mongrand S, McLachlin DT, Chait BT, Chua NH (2002). ABI5 acts downstream of ABI3 to execute an ABA-dependent growth arrest during germination.. Plant J.

[53] Gampala SS, Kim TW, He JX, Tang W, Deng Z (2007). An essential role for 14-3-3 proteins in brassinosteroid signal transduction in Arabidopsis.. Dev Cell.

[54] Ryu H, Kim K, Cho H, Park J, Choe S (2007). Nucleocytoplasmic shuttling of BZR1 mediated by phosphorylation is essential in Arabidopsis brassinosteroid signaling.. Plant Cell.

[55] Valenzuela-Fernandez A, Cabrero JR, Serrador JM, Sanchez-Madrid F (2008). HDAC6: a key regulator of cytoskeleton, cell migration, and cell-cell interactions.. Trends Cell Biol.

[56] Kao HY, Lee CH, Komarov A, Han CC, Evans RM (2002). Isolation and characterization of mammalian HDAC10, a novel histone deacetylase.. J Biol Chem.

[57] Seigneurin-Berny D, Verdin A, Curtet S, Lemercier C, Garin J (2001). Identification of the components of the murine histone deacetylase 6 complex: link between acetylation and ubiquitination signaling pathways.. Mol Cell Biol.

[58] Grozinger CM, Hassig CA, Shreiber SL (1999). Three proteins define a class of human histone deacetylases related to yeast Hda1.. Proc Natl Acad Sci USA.

[59] Guardiola AR, Yao TP (2002). Molecular cloning and characterization of a novel histone deacetylase HDAC10.. J Biol Chem.

[60] Yamamoto K, Hamada H, Shinkai H, Kohno Y, Koseki H (2003). The KDEL receptor modulates the endoplasmic reticulum stress response through mitogen- activated protein kinase signaling cascades.. J Biol Chem.

[61] McKinsey TA, Zhang CL, Lu J, Olson EN (2000). Signal-dependent nuclear export of a histone deacetylase regulates muscle differentiation.. Nature.

[62] Mi HK, Puglielli L (2009). Two Endoplasmic Reticulum (ER)/ER Golgi intermediate compartment-based lysine acetyltransferases post-translationally regulate BACE1 levels.. J Biol Chem.

[63] Gregoretti IV, Lee YM, Goodson HV (2004). Molecular evolution of the histone deacetylase family: functional implications of phylogenetic analysis.. J Mol Biol.

[64] Linseman DA, Bartley CM, Le SS, Laessig TA, Bouchard RJ (2003). Inactivation of the myocyte enhancer factor-2 repressor histone deacetylase-5 by endogenous Ca_2+_/calmodulin-dependent kinase II promotes depolarization-mediated cerebellar granule neuron survival.. J Biol Chem.

[65] van der Linden AM, Nolan KM, Sengupta P (2007). KIN-29 SIK regulates chemoreceptor gene expression via an MEF2 transcription factor and a Class II HDAC.. EMBO J.

[66] Berdeaux R, Goebel N, Banaszynski L, Takemori H, Wandless T (2007). SIK1 is a class II HDAC kinase that promotes survival of skeletal myocytes.. Nat Med.

[67] Vega RB, Harrison BC, Meadows E, Roberts CR, Papst PJ (2004). Protein kinases C and D mediate agonist-dependent cardiac hypertrophy through nuclear export of histone deacetylase 5.. Mol Cell Biol.

[68] Parra M, Kasler H, McKinsey TA, Olson EN, Verdin E (2005). Protein kinase D1 phosphorylates HDAC7 and Induces its nuclear export after TCR activation.. J Biol Chem.

[69] Dequiedt F, Van Lint J, Lecomte E, Van Duppen V, Seufferlein T (2005). Phosphorylation of histone deacetylase 7 by protein kinase D mediates T cell receptor- induced Nur77 expression and apoptosis.. J Exp Med.

[70] Martin M, Kettmann R, Dequiedt F (2007). Class IIa histone deacetylases: regulating the regulators.. Oncogene.

[71] Chang S, Bezprozvannaya S, Li S, Olson EN (2005). An expression screen reveals modulators of class II histone deacetylase phosphorylation.. Proc Natl Acad Sci USA.

[72] Kim MA, Kim HJ, Brown AL, Lee MY, Bae YS (2007). Identification of novel substrates for human checkpoint kinase Chk1 and Chk2 through genome-wide screening using a consensus Chk phosphorylation motif.. Exp Mol Med.

[73] Blander G, Guarente L (2004). The Sirt2 family of protein deacetylases.. Annu Rev Biochem.

[74] Schwer B, North BJ, Frye RA, Ott M, Verdin E (2002). The human silent information regulator (Sir)2 homologue hSIRT3 is a mitochondrial nicotinamide adenine dinucleotide-dependent deacetylase.. J Cell Biol.

[75] Wallace DC (2010). The epigenome and the mitochondrion: bioenergetics and the environment.. Genes Dev.

[76] Wallace DC, Fan W (2010). Energetics, epigenetics, mitochondrial genetics.. Mitochondrion.

[77] Chua YL, Watson LA, Gray JC (2003). The transcriptional enhancer of the pea plastocyanin gene associates with the nuclear matrix and regulates gene expression through histone acetylation.. Plant Cell.

[78] Sheldon CC, Finnegan EJ, Dennis ES, Peacock WJ (2006). Quantitative effects of vernalization on FLC and SOC1 expression.. Plant J.

[79] Chen ZJ, Tian L (2007). Roles of dynamic and reversible histone acetylation in plant development and polyploidy.. Biochim Biophys Acta.

[80] Clough SJ, Bent AF (1998). Floral dip: A simplified method for Agrobacterium- mediated transformation of *Arabidopsis thaliana*.. Plant J.

[81] Cutler S, Ehrhardt DW, Griffitts JS, Somerville CR (2000). Random GFP:cDNA fusions enable visualization of subcellular structures in cells of Arabidopsis at high frequency.. Proc Natl Acad Sci USA.

[82] Yoo SD, Cho YH, Sheen J (2007). Arabidopsis mesophyll protoplasts: a versatile cell system for transient gene expression analysis.. Nat Protoc.

[83] Miao Y, Jiang L (2007). Transient expression of fluorescent fusion proteins in protoplasts of suspension cultured cells.. Nat Protocols.

[84] Lee LY, Fang MJ, Kuang LY, Gelvin SB (2008). Vectors for multi-color bimolecular fluorescence complementation to investigate protein-protein interactions in living plant cells.. Plant Methods.

[85] Nelson BK, Cai X, Nebenfuhr A (2007). A multicolored set of in vivo organelle markers for co-localization studies in Arabidopsis and other plants.. Plant J.

[86] Ryu H, Kim K, Cho H, Park J, Choe S (2007). Nucleocytoplasmic shuttling of BZR1 mediated by phosphorylation is essential in Arabidopsis brassinosteroid signaling.. Plant Cell.

[87] Yanagisawa S, Yoo SD, Sheen J (2003). Differential regulation of EIN3 stability by glucose and ethylene signaling in plants.. Nature.

[88] Sohn EJ, Kim ES, Zhao M, Kim SJ, Kim H (2003). Rha1, an Arabidopsis Rab5 homolog, plays a critical role in the vacuolar trafficking of soluble cargo proteins.. Plant Cell.

[89] Lu Q, Tang X, Tian G, Wang F, Liu K (2010). Arabidopsis homolog of the yeast TREX-2 mRNA export complex: components and anchoring nucleoporin.. Plant J.

