# Supplementary figures and images for: Subcellular Localization of Class II HDAs in Arabidopsis thaliana: Nucleocytoplasmic Shuttling of HDA15 Is Driven by Light
Source: PLoS One. 2012 Feb 17;7(2):e30846. doi: 10.1371/journal.pone.0030846 (PMC3281883; doi:10.1371/journal.pone.0030846)

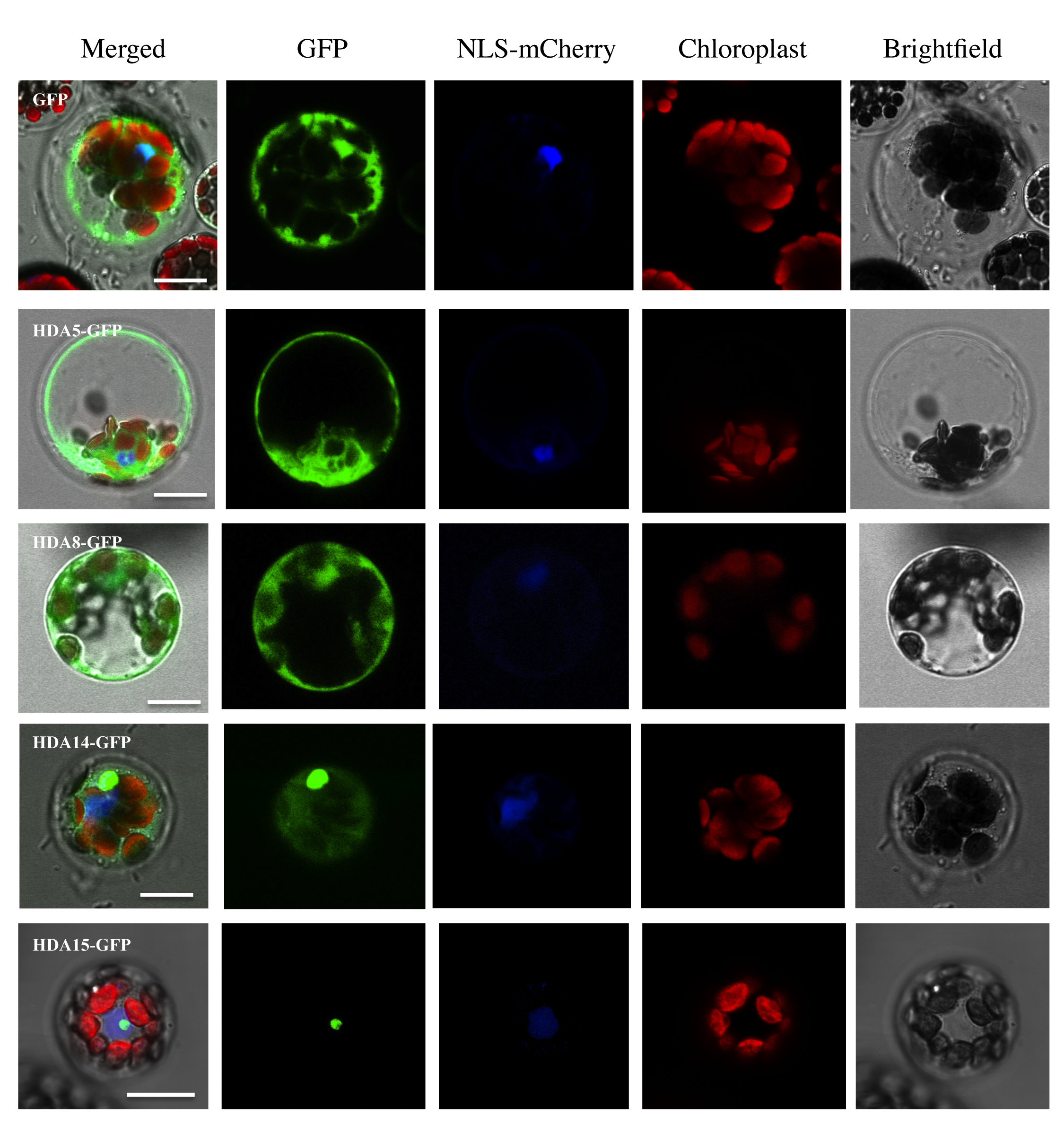

Supplement: Figure S1 — Protoplast transient expression using GFP-HDA fusion constructs. Subcellular localization of Class II HDAs was determined via protoplast PEG transfection using GFP-HDA fusion constructs. HDA5, HDA8, and HDA14 were cytoplasmic while HDA15 was restricted inside the nucleus. VirD2NLS fused with mCherry was used as a nuclear marker. Scale bars were calibrated to 10 µm. (TIF) [file pone.0030846.s001.tif]

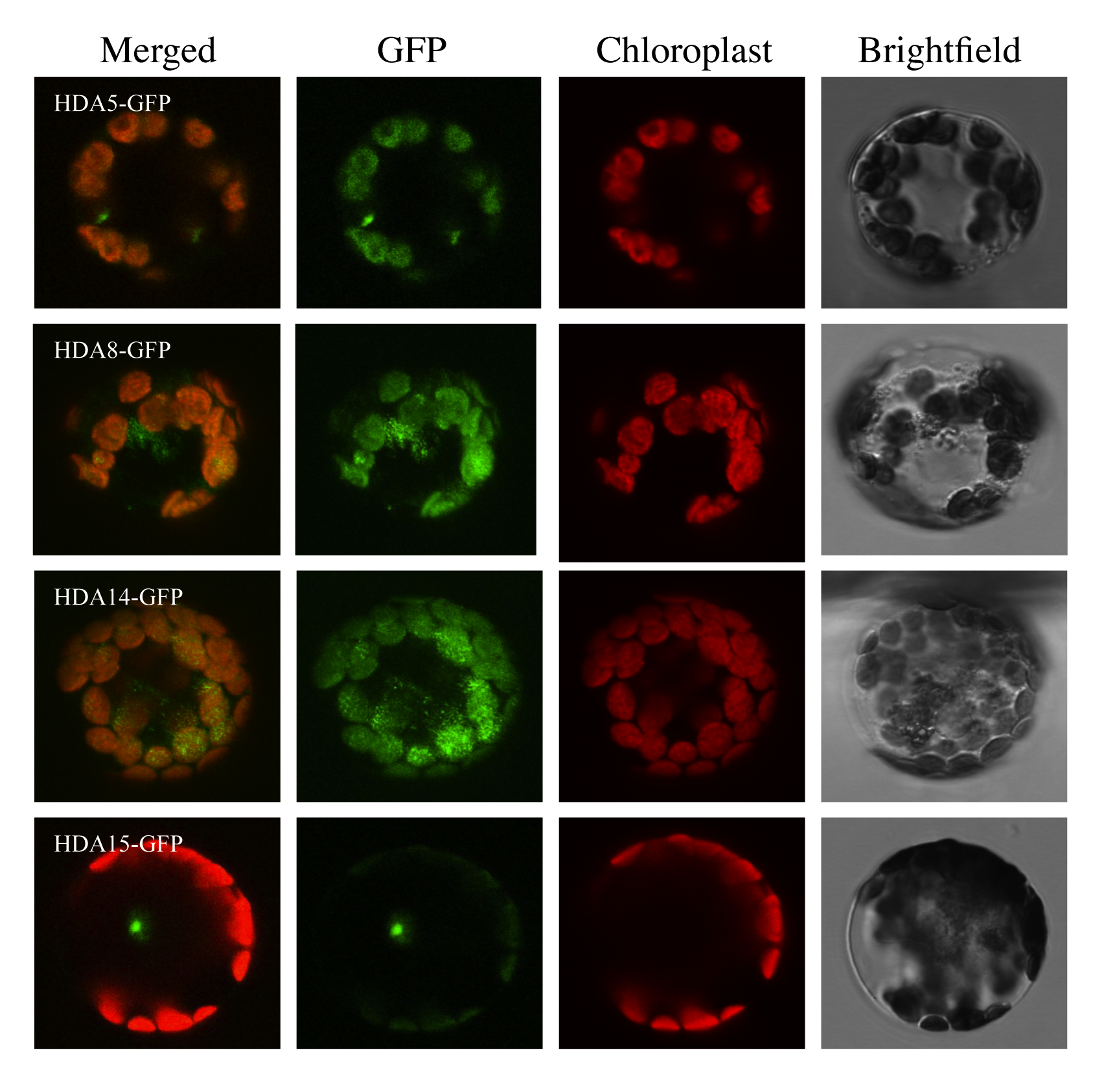

Supplement: Figure S2 — Subcellular localization in transgenic protoplasts. Protoplasts from transgenic lines of Class II HDAs were also used to determine their corresponding subcellular localization. Although GFP signals were relatively weak, HDA5, HDA8, and HDA14 were found to abound in the cytoplasm while HDA15 emanated strong nuclear signals. (TIF) [file pone.0030846.s002.tif]

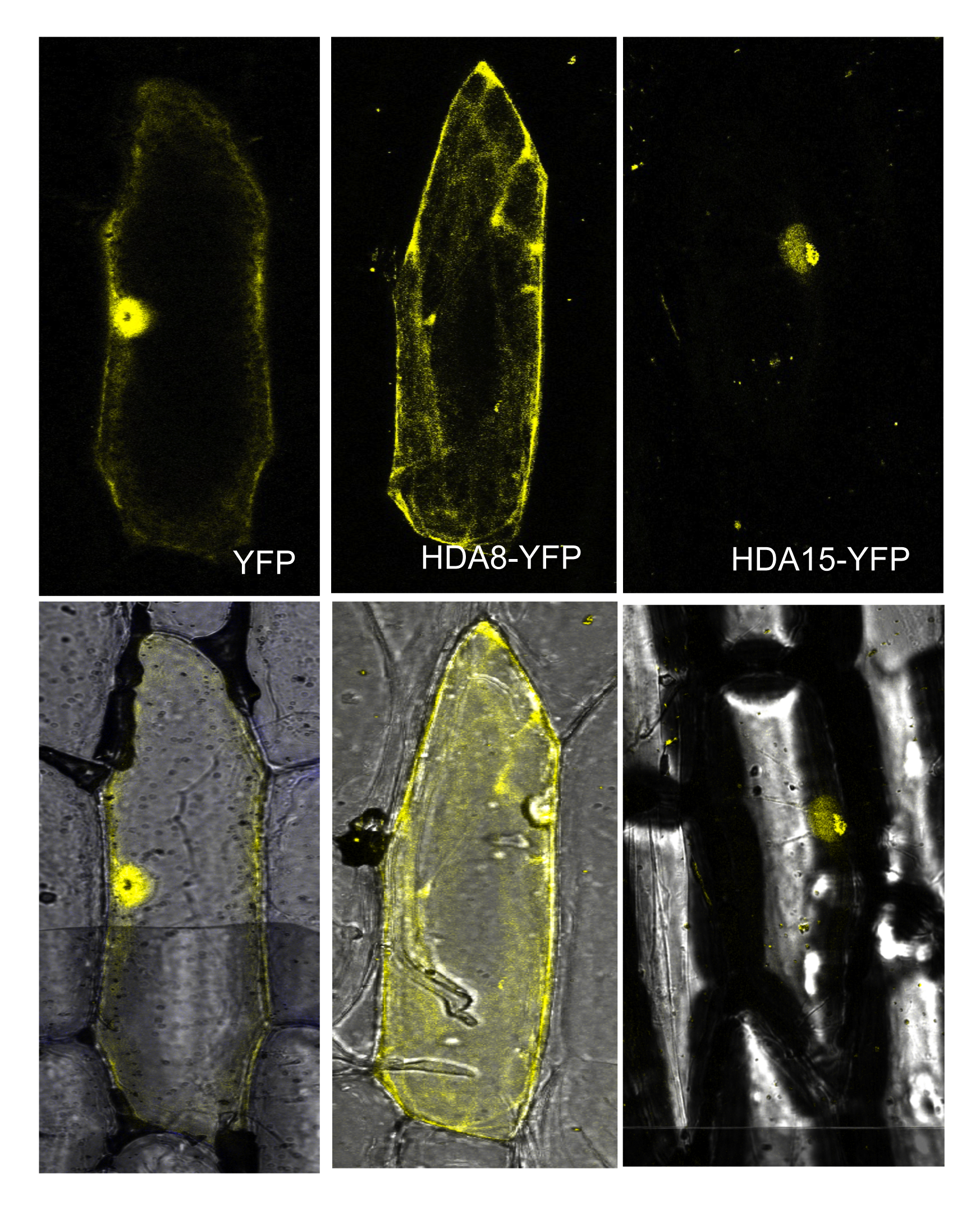

Supplement: Figure S3 — Particle bombardment in onion tissues. Overlay pictures reveal the nuclear and cytoplasmic localization of HDA8-YFP and the nuclear concentration of HDA15. (TIF) [file pone.0030846.s003.tif]

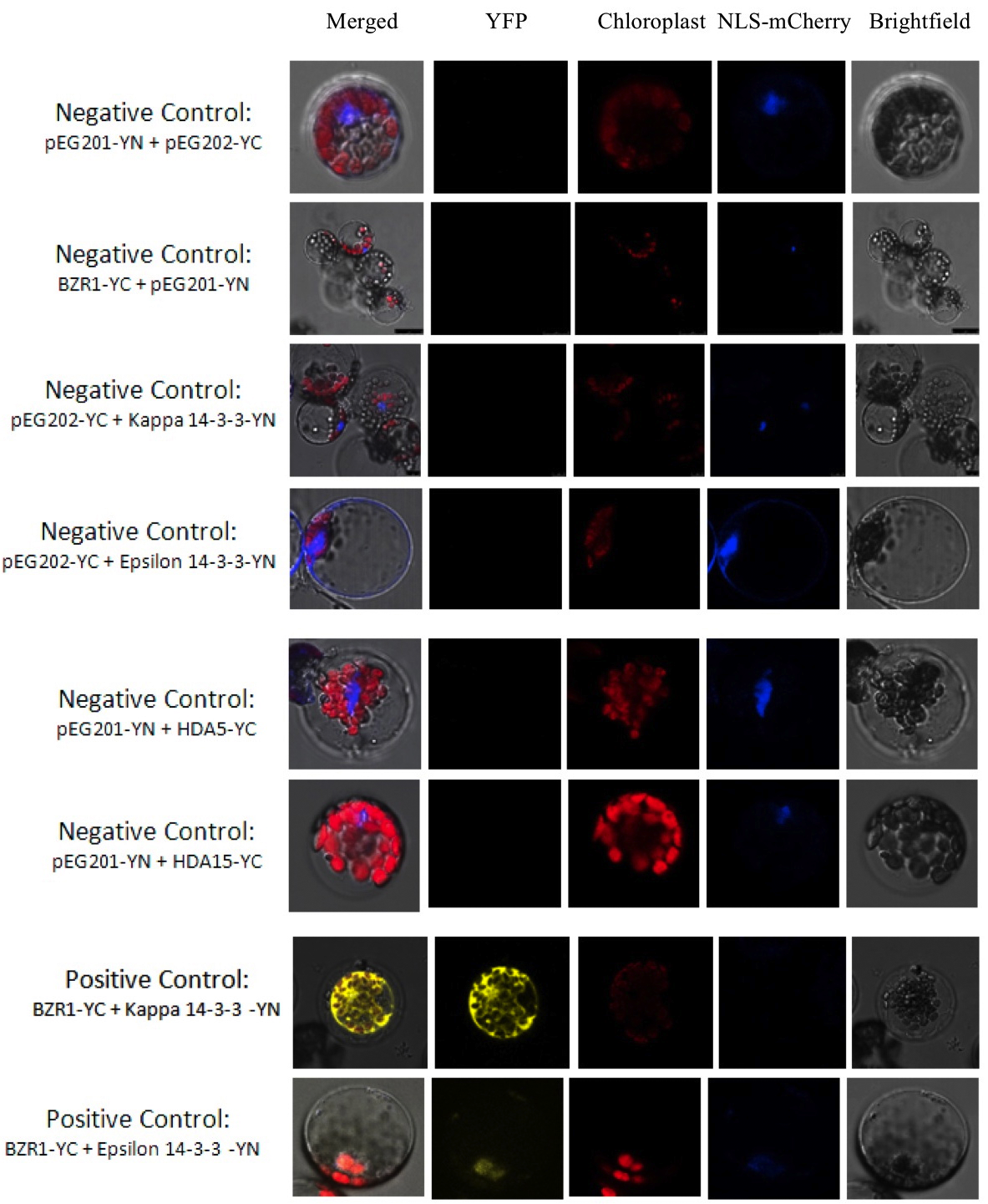

Supplement: Figure S4 — BiFC negative and positive controls. Empty vectors and YN/YC constructs were tested as negative controls. BZR1 was used as a positive control for the 14-3-3 kappa and epsilon interactions. (TIF) [file pone.0030846.s004.tif]

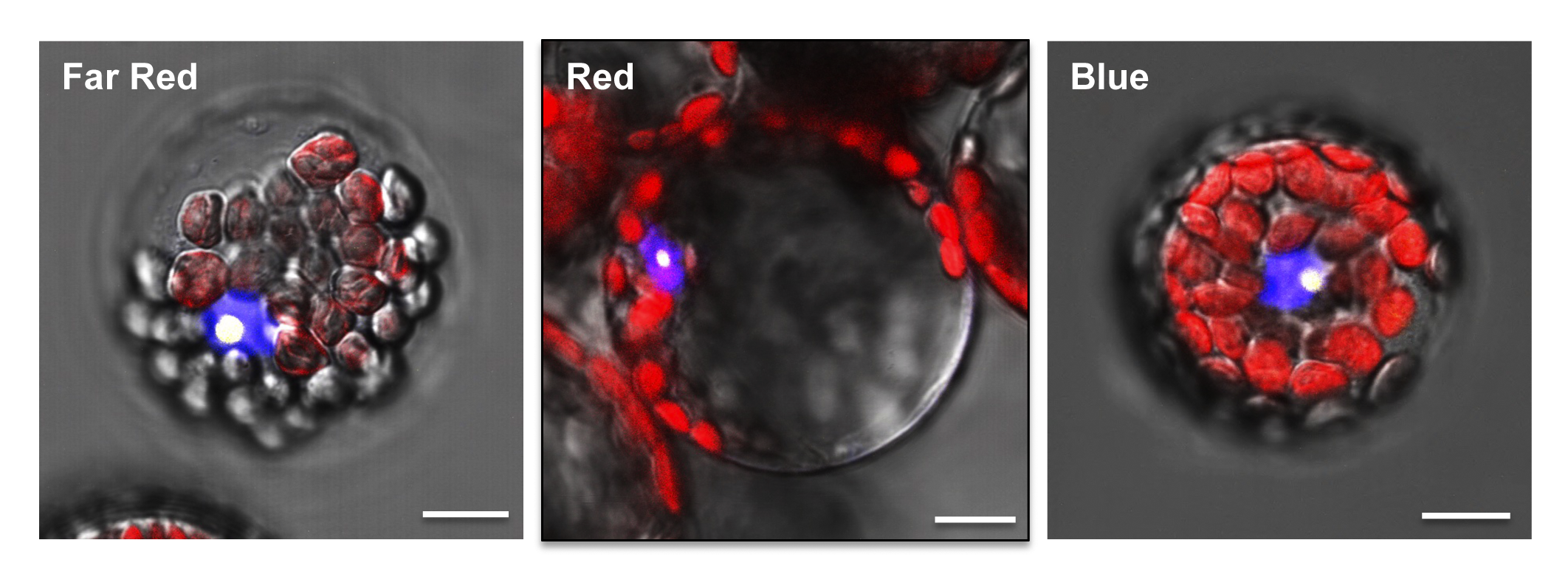

Supplement: Figure S5 — Nucleolar localization of HDA15-YFP in different light treatments. Transfected protoplasts were incubated under white light for 18 h then transferred to far red, red, and blue light treatments for 3 h at low light intensities (FR 2.77 µmol m−2 s−1, R 1.77 µmol m−2 s−1. B 3.84 µmol m−2 s−1). Similar with white light treated protoplasts, HDA15-YFP was restricted in a small spot inside the nucleus, potentially nucleolus. VirD2NLS-mCherry was co-transfected as a nuclear marker (blue). Scale bars were calibrated to 10 µm. (TIF) [file pone.0030846.s005.tif]
